# Syndesmosis Changes before and after Syndesmotic Screw Removal: A Retrospective Radiographic Study

**DOI:** 10.3390/medicina58030445

**Published:** 2022-03-18

**Authors:** Chien-Ting Huang, Peng-Ju Huang, Cheng-Chang Lu, Chia-Lung Shih, Yuh-Min Cheng, Shu-Jung Chen

**Affiliations:** 1 School of Post-Baccalaureate Medicine, Kaohsiung Medical University, Kaohsiung 807, Taiwan; neverstop1211@gmail.com; 2 Department of Orthopedics, Kaohsiung Medical University Hospital, Kaohsiung 807, Taiwan; roger01@ms4.hinet.net (P.-J.H.); cclu0880330@gmail.com (C.-C.L.); stone770116@gmail.com (C.-L.S.); 3 Department of Orthopedics, Kaohsiung Municipal Siaogang Hospital, Kaohsiung 812, Taiwan; 4 Regenerative Medicine and Cell Therapy Research Center, Kaohsiung Medical College, Kaohsiung 807, Taiwan; 5 Department of Orthopedics, Pingtung Hospital, Pingtung City 900, Taiwan; 0710058@pntn.mohw.gov.tw

**Keywords:** ankle fracture, diastasis, screw removal, syndesmosis, syndesmotic screw

## Abstract

Background and Objectives: In patients with ankle fractures complicated by syndesmotic injuries, no consensus has been reached on the best method of syndesmosis fixation using syndesmotic screws. One previous study revealed no difference in the tibiofibular overlap between two groups with or without syndesmotic screw removal. Other studies have indicated that distal tibiofibular diastasis exists after the removal of syndesmotic screws. In this study, we aimed to confirm the effect of syndesmotic screw removal on diastasis occurrence. We further analyzed the risk factors that may contribute to the widening of the tibiofibular syndesmosis. Materials and Methods: This retrospective study involved a review of the records of 63 patients with ankle fractures accompanied by syndesmosis injuries that required syndesmotic screw fixation. Anteroposterior radiographs were analyzed for each patient at various time points, from syndesmotic screw fixation to outpatient department follow-ups after screw removal. The changes in tibia–fibula overlap (OL), tibia–fibula clear space (CS), and medial clear space (MCS) were analyzed. Further analysis was performed to reveal potential factors that may have contributed to radiographic differences. Results: Compared with the postoperation radiographs following syndesmotic screw fixation, OL decreased (2.0 mm) and CS increased (0.8 mm) in the anteroposterior radiographs from outpatient department follow-ups. No significant changes were noted in OL or CS after syndesmotic screw removal. However, OL decreased (1.8 mm) and CS increased (0.5 mm) before syndesmotic screw removal. No significant change in MCS occurred during the whole observation period. Linear regression analysis did not reveal any significant correlations between potentially related factors and radiographic changes. Conclusions: Marked diastasis had occurred at final follow-up. Notably, the diastasis occurred before rather than after screw removal. This implies that screw removal does not significantly influence the radiographic outcomes of rotational ankle fractures.

## 1. Introduction

Patients of ankle fractures with syndesmotic injuries are prone to postoperative diastasis, incongruity of the fibula within the incisure, and posterior translation of the talus [1,2]. However, treatments for syndesmotic injuries vary, and trans-syndesmotic fixation using screws is among the most common treatments in clinical practice.

No consensus has been reached on the best practices of trans-syndesmotic fixation using screws in terms of the selection of the syndesmotic screws, the size of the syndesmotic screws, and the timing of screw removal. Screw removal is still debated due to the uncertainty of its effects on posttraumatic patients with syndesmosis injuries. One study reported that 1 year postsurgery, no statistically significant differences were observed in the tibiofibular clear space between patients with or without syndesmotic screw removal [3]. Another study also reported no significant differences in the mean radiological tibiofibular clear space between two groups with or without syndesmotic screw removal [4]. However, some studies revealed the presence of distal tibiofibular diastasis after syndesmotic screw removal [5,6,7].

In this study, we aimed to confirm whether diastasis occurred after syndesmotic screw removal and analyze the risk factors related to diastasis of the distal tibiofibular syndesmosis. This study mainly focused on the association between syndesmotic screw removal and radiological changes in the ankles

## 2. Materials and Methods

We retrospectively reviewed all the records of patients with ankle fractures from January 2015 through December 2018 at our hospital. We included patients who (1) sustained Lauge–Hansen classification supination external rotation (SER) or pronation external rotation (PER) ankle fractures, (2) received open reduction and internal fixation of the ankle fracture with syndesmotic screw fixation less than 2 weeks postinjury, and (3) underwent scheduled trans-syndesmotic screw removal. Patients were excluded if they had an open fracture or pilon fracture, if they had a high risk of skin complication, or if they did not undergo syndesmotic screw removal due to advanced age, underlying medical conditions, or personal preferences.

### 2.1. Operative Technique and Rehabilitation Protocol

Patients underwent operations under anesthesia in the supine position. All the malleolar fractures were reduced and fixed using plates or screws according to the AO Foundation standards. After fixation of the fibula, medial malleolus, and posterior malleolus, syndesmosis stability was assessed based on a lateral stress test or an external rotation stress test using direct visualization or fluorography. Syndesmotic screw fixation was applied in patients with syndesmotic instability or syndesmotic malposition, as determined by preoperational imaging or intra-operative assessment. After reduction, the syndesmotic screws were inserted through four cortices.

Ankle range of motion (ROM) exercise and toe touch weight-bearing (TTWB) ambulation were initiated immediately after surgery and continued for 6 weeks. Then, if the radiographic outcomes were acceptable at first-time follow-up around post-operative 6 weeks, partial to full weight-bearing ambulation was instructed in a stepwise manner. Removal of syndesmotic screws was routinely arranged after full weight-bearing on the affected ankle was achieved, around post-operative 10 to 12 weeks. All included patients followed similar rehabilitation protocols without major complications or drop out.

### 2.2. Radiographic Evaluation

Radiographs, which were displayed through Digital Imaging and Communication in Medicine, were retrospectively reviewed for each patient. The radiographs, which were taken pre-operatively, post-operatively, before removal of the screws and at final follow-up, were collected and assessed. All records were examined by a fourteen-year-experienced attending physician (SJC). Intraclass coefficients (ICC) were also analyzed to validate intra-rater reliability. Fracture type was categorized using the Lauge–Hansen classification system.

**Figure 1:**
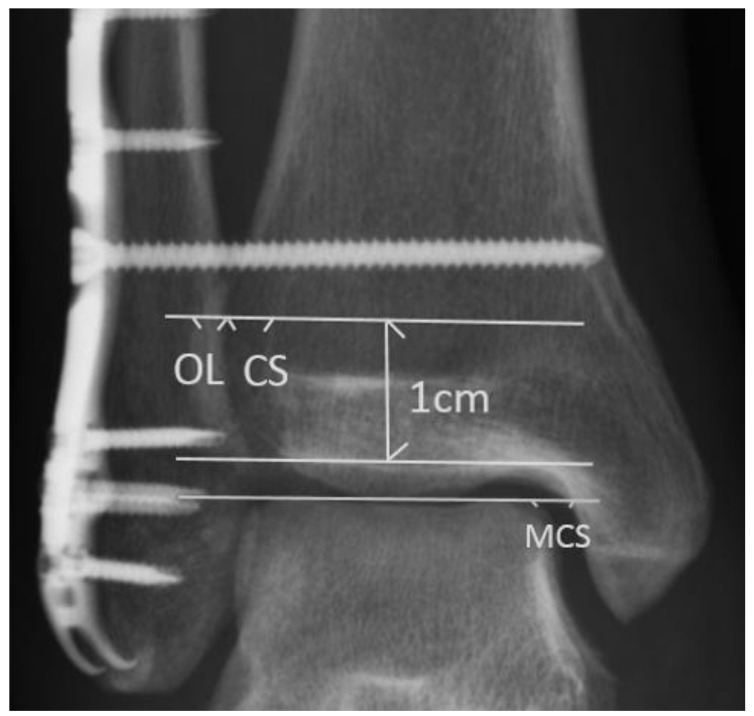
Radiographic evaluation in the study. The tibiofibular clear space (CS) was defined as the distance between the lateral border of the posterior tibial malleolus and the medial aspect of the fibula, measured 1 cm proximal to the tibial plafond. The medial clear space (MCS) was defined as the distance from the lateral border of the medial malleolus to the medial border of the talus at the level of the talar dome. The tibiofibular overlap (OL) was measured from the lateral border of the anterior tibial prominence to the medial fibula 1 cm proximal to the tibial plafond.

For each standard ankle anteroposterior (AP) radiograph, tibiofibular overlap (OL), tibiofibular clear space (CS), and medial clear space (MCS) were measured. The OL was defined as the distance between the lateral border of the anterior tibial prominence and the medial fibula 1 cm proximal to the tibial plafond [8]. The CS was defined as the distance between the lateral border of the posterior tibial malleolus and the medial aspect of the fibula measured 1 cm proximal to the tibial plafond [8]. The MCS was defined as the distance from the lateral border of the medial malleolus to the medial border of the talus at the level of the talar dome (Figure 1) [8]. A radiolucent zone is always found between the implant and the surrounding bone, usually parallel to the implant surface. In our study, we defined the positive radiolucent line as a radiolucent zone more than half of the syndesmosis screw.

**Figure 2:**
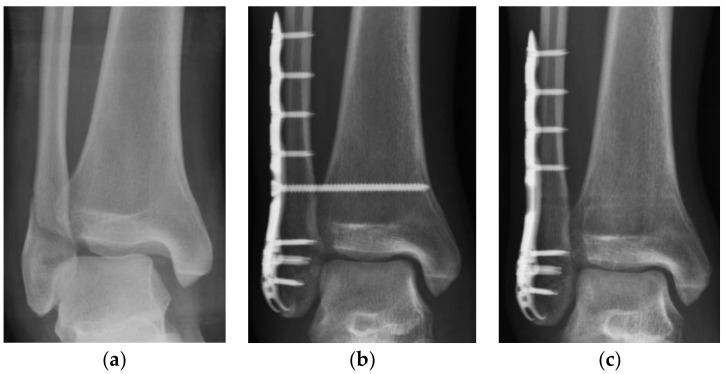
Demonstration of serial images evaluation: (**a**) Patients who met the inclusion criteria were collected; (**b**) pre-SR anteroposterior radiographs were obtained; (**c**) last-FU anteroposterior radiographs were obtained. CS: tibiofibular clear space; last-FU: last follow-up; OL: tibiofibular overlap; pre-SR: presyndesmotic screw removal.

Data analysis of CS, OL, and MCS in anteroposterior radiographs was conducted at three time points: after syndesmotic fixation (post-SF), before syndesmotic screw removal (pre-SR), and at the last follow-up (last-FU). The post-SF radiographs were obtained immediately after syndesmotic fixation. The pre-SR radiographs were obtained immediately before the syndesmotic screws were removed. The last-FU radiographs were the most recent follow-up radiographs that could be obtained after the syndesmotic screws were removed. To compare the difference among the radiographic outcomes at each time point, three periods of interest were defined. The total observation period was defined as the period from the post-SF time point to the last-FU time point. The pre-SR period was defined as the period from the post-SF time point to the pre-SR time point and was equal to the duration of syndesmotic screw retention. The post-SR period was defined as the period from the pre-SR time point to the last-FU time point (Figure 2).

### 2.3. Statistical Analysis

The data were analyzed using SAS version 9.4 for Windows (SAS Inc., Taipei, Taiwan). A paired-samples *t*-test was used to analyze the radiographic outcomes of the pre-SR period, post-SR period, and total observation period. Further analysis focused on the significant differences revealed by the paired samples *t*-test. An analysis of possible related factors (age, gender, body mass index (BMI), length of pre-SR period, fracture type (SER or PER; with dislocation or without dislocation), and the radiolucent zone around the syndesmotic screws) was performed using a linear regression model to evaluate the associations between radiographic outcomes and each respective factor. Relationships were considered statistically significant at *p* < 0.05.

## 3. Results

### 3.1. Participants

Sixty-three patients (twenty-two men, forty-one women) with an average age of 46.2 years (range, 15–78 years) who underwent syndesmotic screw fixation were included in this study. The average body mass index of the patients was 25.77 ± 4.73 (range, 17.10 to 39.19). Of the indicated injuries, 46 (63.1%) were SER injuries and 17 (36.9%) were PER injuries. Nine of the patients (14.3%) had indicated injuries with dislocation. Syndesmosis fixation was performed with one 3.5 mm syndesmotic screw in all study participants (Table 1). In the pre-SR period, 14 (22.2%) of the patients had syndesmotic screws with a radiolucent zone.

### 3.2. Main Results

Standard ankle radiographs were evaluated from three time periods: the total observation period, pre-SR period, and post-SR period. In the total observation period, OL decreased by an average of 2.0 mm ± 2.8 mm (range, −10.1 to 5.0 mm; *p* < 0.001), CS increased by an average of 0.8 mm ± 1.3 mm (range, −1.8 to 5.8 mm; *p* < 0.001), and MCS increased by an average of 0.1 mm ± 1.3 mm (range, −2.8 to 3.6 mm; *p* = 0.495). The changes in OL and CS in the total observation period were statistically significant. No significant change in MCS in the total observation period was observed. (Table 2).

The duration of the pre-SR period was 10.0 ± 0.4 weeks (Table 1). During this period, OL decreased by an average of 1.8 ± 2.7 mm (range, −10.0 to 4.7 mm; *p* < 0.001). CS increased by an average of 0.5 ± 1.5 mm (range, −3.0 to 7.3 mm; *p* = 0.015), and MCS decreased by an average of 0.1 ± 1.2 mm (range, −2.7 to 5.0 mm; *p* = 0.442). The changes in the OL and CS in the pre-SR period were also significant. No significant changes were observed in the MCS (Table 3).

The duration of the post-SR period was 10.9 ± 1.2 months. During this period, OL decreased by an average of 0.3 ± 2.0 mm (range, −7.1 to 5.1 mm; *p* = 0.432), CS increased by an average of 0.3 ± 1.0 mm (range, −1.8 to 3.6 mm; *p* = 0.054), and MCS increased by an average of 0.2 mm ± 0.9 (range, −1.9 to 3.4 mm; *p* = 0.106). However, the changes in OL, CS, or MCS in this period were not statistically significant (Table 4).

Intra-rater reliability was evaluated, with an average ICC 0.88 ± 0.06 (range, 0.76 to 0.96) for each parameter analyzed in this study.

A pre-SR period analysis was performed to determine the correlation between the radiographic outcomes and the following factors: age, gender, BMI, duration of the pre-SR period, fracture type (SER or PER; with dislocation or without dislocation), and radiolucent zone around the syndesmotic screws. The analysis did not reveal any significant correlations between the radiographic outcomes and the potential influencing factors (Table 5). Additionally, no recurrent ankle subluxations or dislocations were observed in serial radiographic surveys.

## 4. Discussion

In this study, we found a reduction in OL and an increase in CS in the total observation period. This indicates that syndesmotic diastasis occurred between the placement of the syndesmotic screw and the final follow-up after screw removal. The period above was defined as the total observation period and was divided into a pre-SR period and a post-SR period. Surprisingly, diastasis was observed in the pre-SR period rather than in the post-SR period. This finding indicates that although diastasis was observed at final follow-up, it occurred before screw removal rather than after screw removal. These results are noteworthy and differ from the results of other studies.

Endo et al. used computer tomography to evaluate syndesmotic reduction 2 weeks after syndesmotic screw fixation and 1 year after screw removal [9]. They found that the anterior distance from the tibia to the fibula was significantly longer 1 year after screw removal compared with at 2 weeks after syndesmotic screw fixation. Our findings were in line with the findings of the study by Endo et al. in that diastasis occurred, but they did not clarify whether diastasis occurred before or after screw removal [9]. Boyle et al. compared patients who did not undergo screw removal to those who underwent syndesmotic screw removal 1 year after syndesmotic screw fixation [7]. The study reported no significant differences in the tibiofibular clear space between the two groups. However, radiographs were only evaluated 1 year after syndesmotic screw fixation; no radiographic comparisons could be made of a patient’s condition before and after screw removal. Therefore, on the basis of the results of our study, we postulate that diastasis occurred before the removal of syndesmotic screws, and no significant syndesmotic space changes occurred after screw removal.

In the study by Jordan et al., all participants underwent screw removal 11 to 16 weeks after syndesmotic fixation, and diastasis was noted after screw removal [5]. This trend differs from our results, which indicate that diastasis occurred in the pre-SR period. The contrast between these findings may be related to the difference in rehabilitation protocols. In the study by Jordan et al., patients were placed in a below-knee cast after the removal of skin sutures and continued non-weight-bearing ambulation for 6 weeks. Only patients with low-energy rotation injuries were permitted to bear weight on the affected ankle, and all patients were allowed to bear weight while wearing a removable boot after screw removal. In our study, all patients began TTWB and ankle ROM exercises immediately after syndesmotic fixation; the aim of this protocol was to prevent the restriction of ankle ROM and to counter potential decreases in muscle power and post-operative functional outcomes. Needleman et al. concluded that removing syndesmotic screws was necessary and that non-weight-bearing exercises should be performed before screw removal [10]. Moore et al. also observed greater loss of reduction when the patients were not compliant with weight-bearing restriction for 6 weeks post-operation [11]. In our study, patients undertook ankle ROM and TTWB exercises beginning immediately after surgery and continuing for 6 weeks. Then, in the following 6 weeks, partial to full weight-bearing was advised in a stepwise manner. The differences between our study and other articles are rehabilitation protocol and time to weight-bearing. Therefore, rehabilitation protocols may influence the occurrence of diastasis according to our results and previous studies. It implies that a little diastasis occurs after repeated body weight applying to the ankle joint.

Jordan et al. also found that the MCS did not change significantly in spite of fibula widening [5]. They concluded that although diastasis occurred, the ankle joint remained stable. This result corresponded to our finding of no significant change in the MCS during the entire observational period.

We subsequently investigated potential risk factors that may have been correlated with ankle diastasis during the fixation period. An analysis of correlation was performed between radiographic outcomes and the following potential risk factors: age, gender, BMI, duration of the pre-SR period, fracture type (SER or PER; with dislocation or without dislocation), and the radiolucent zone around the syndesmotic screws. According to the results, none of the potential factors were significantly correlated with the radiographic outcomes of the patients (Table 5). Although more patients with PER fractures underwent syndesmotic screw fixation than did patients with SER fractures, the frequency of diastasis was not significantly different between the two groups. This may be explained by the fact that fractures requiring syndesmotic screw fixation all involved syndesmotic injuries, regardless of fracture type. Furthermore, although we predicted BMI to be a risk factor of diastasis, our analysis did not reveal a significant correlation. Future analyses of correlations should include activity level and assistance level as potential risk factors. Because we did not find any significantly correlated risk factors in this analysis, it is still unclear why diastasis occurred.

This study had several limitations. First, incomplete radiograph follow-up records may have led to unrepresentative samples, despite the loss of only one data set in the pre-SR period and post-SR period. Nonetheless, the dataset that we collected was sufficient to detect statistically significant differences. Second, we only used standard ankle anteroposterior plain films to investigate the syndesmotic condition. Baek et al. suggested that computed tomography CT should be used to confirm syndesmosis malreduction [12]. However, although plain films are not as accurate as CT, they are a common and convenient tool without extra radiation. Most other previous studies have used this method to evaluate syndesmosis, and such results have been easy to apply in clinical practice. Additionally, we demonstrated the difference in radiographic outcomes between the pre-SR, post-SR, and total observation periods. Using serial comparison of radiographs, we demonstrated the changes in syndesmotic fixation for each period. Third, the present study was a retrospective article, not a prospective one, and the follow-up period was not very long. Moreover, we decided to evaluate radiography by a single experienced physician to eliminate inter-rater variability, and the intra-rater reliability was good. However, the lack of multiple raters might cause bias and was the weakness of our study. Finally, we did not measure functional outcomes, and therefore we cannot confirm the relationship between the radiographic findings and functional performance. One year after syndesmotic screw fixation, Boyle et al. [3] did not observe any differences in functional outcomes between groups with and without syndesmotic screw removal. However, without comparing the functional outcomes before and after screw removal, we cannot confirm whether a difference existed in the functional scores of patients who underwent syndesmotic screw removal. Therefore, further investigation into the associations between radiographic and functional outcomes is warranted.

## 5. Conclusions

In this study, we observed that diastasis had occurred at the final follow-up after syndesmotic screw removal. Notably, the diastasis occurred before screw removal rather than after screw removal. This implies that the removal of the syndesmotic screw does not significantly influence the radiographic outcomes of rotation ankle fractures.

## Figures and Tables

**Table 1:** Patient characteristics.

Total *N*	
Male	22 (34.9%)
Female	41 (65.1%)
Age, y	
Mean ± SD	46.2 ± 17.6
Body mass index	25.77 ± 4.73
Mean ± SD	
Fracture type	
SER	46 (63.1%)
PER	17 (36.9%)
Complicated with dislocation	9 (14.3%)
No. of Screws	1 screw
Screw size, mm	3.5
Syndemotic screw with radiolucent zone	14 (22.2%)
pre-SR period duration, wk	
Mean ± SD	10.0 ± 0.4
post-SR period duration, mth	
Mean ± SD	10.9 ± 1.12

Pre-SR: presyndesmotic screw removal; post-SR: postsyndesmotic screw removal; PER: pronation-external rotation; SER: supination-external rotation; SD: standard deviation.

**Table 2:** Differences in radiographic outcomes for the total observation period.

Variable	*N*	Mean (Minimum–Maximum)	Std Dev	*p*-Value
OL change, mm	63	−2.0 (−10.1–5.0)	2.8	*p* < 0.001
CS change, mm	63	0.8 (−1.8–5.8)	1.3	*p* < 0.001
MCS change, mm	63	0.1 (−2.8–3.6)	1.3	0.495

Radiographs of the total observation period were analyzed using paired samples *t*-test. CS: tibiofibular clear space; MCS: medial clear space; OL: tibiofibular overlap; Std Dev: standard deviation.

**Table 3:** Differences in radiographic outcomes for the pre-SR period.

Variable	*N*	Mean (Minimum–Maximum)	Std Dev	*p*-Value
OL change, mm	62	−1.8 (−10.0–4.7)	2.7	*p* < 0.001
CS change, mm	62	0.5 (−3.0–7.3)	1.5	0.015 (*p* < 0.05)
MCS change, mm	62	−0.1 (−2.7–5.0)	1.2	0.442

Radiographs from the pre-SR period were analyzed using a paired samples *t*-test. CS: tibiofibular clear space; MCS: medial clear space; OL: tibiofibular overlap; pre-SR: presyndesmotic screw removal; Std Dev: standard deviation.

**Table 4:** Differences in radiographic outcomes for the post-SR period.

Variable	*N*	Mean (Minimum–Maximum)	Std Dev	*p*-Value
OL change, mm	62	−0.3 (−7.1–5.1)	2.0	0.432
CS change, mm	62	0.3 (−1.8–3.6)	1.0	0.054
MCS change, mm	62	0.2 (−1.9–3.4)	0.9	0.106

Radiographs of the post-SR period were analyzed using a paired samples *t*-test. CS: tibiofibular clear space; MCS: medial clear space; OL: tibiofibular overlap; post-SR: postsyndesmotic screw removal; Std Dev: standard deviation.

**Table 5:** Analysis of possible factors related to syndesmosis widening in the pre-SR period.

	Variable	β	Standard Error	*t* Value	*p*-Value
OL	Age	0.000464	0.002	0.24	0.812
	Sex (M vs. F)	−0.128	0.070	−1.84	0.070
	Body mass index (BMI)	−0.004	0.007	−0.58	0.563
	days of pre-SR period	−0.006	0.042	−0.14	0.891
	SER vs. PER	0.073	0.079	0.92	0.363
	Fracture w/o dislocation	−0.116	0.073	−1.60	0.114
	Radiolucent zone	−0.015	0.082	−0.19	0.853
CS	Age	−0.0000871	0.001	−0.08	0.936
	Sex (M vs. F)	0.041	0.039	1.04	0.302
	Body mass index (BMI)	0.001	0.004	0.28	0.778
	days of pre-SR period	−0.001	0.023	−0.03	0.975
	SER vs. PER	−0.042	0.042	−0.97	0.336
	Fracture w/o dislocation	0.010	0.041	0.25	0.803
	Radiolucent zone	0.016	0.045	0.35	0.731

Factors that may be associated with syndesmosis widening were listed and analyzed using a linear regression model. BMI: Body mass index; CS: tibiofibular clear space; OL: tibiofibular overlap; pre-SR: presyndesmotic screw removal; PER: pronation-external rotation; SER: supination-external rotation.

## Data Availability

The datasets used and analyzed during the current study are available from the corresponding author on reasonable request.
